# Economic appraisal of using genetics to control *Streptococcus agalactiae* in Nile tilapia under cage and pond farming system in Malaysia

**DOI:** 10.1038/s41598-022-12649-9

**Published:** 2022-05-24

**Authors:** Marina Delphino, Rajesh Joshi, Alejandro Tola Alvarez

**Affiliations:** 1 GenoMar Genetics Group, 105N, Al. das Aroeiras, Lote 4, Ap.1402, Palmas, TO Brazil; 2 GenoMar Genetics Group, Oluf Thesens vei 6, 1433 Ås, Norway; 3 GenoMar Genetics Group, Bolette Brygge 1, 0252 Oslo, Norway

**Keywords:** Genetics, Diseases

## Abstract

Disease outbreaks have been seen as the major threat to sustainable aquaculture worldwide. Injectable vaccines have been one of the few strategies available to control the diseases, however, the adoption of this technology globally is limited. Genetic selection for disease resistance has been proposed as the alternative strategy in livestock and aquaculture. Economic analysis for such strategies is lacking and this study assesses the economic worth of using tilapia fingerlings resistant to Streptococcosis in both cage and pond production systems. The paper also assesses the profitability of paying the higher price for such fingerlings. Partial-budgeting was used to develop a stochastic simulation model that considers the benefits and costs associated with the adoption of tilapia fingerlings resistant to Streptococcosis at the farm level, in one production cycle. In both ponds and cage production systems, the use of genetically selected Streptococcus resistant tilapia fingerlings was found to be profitable where Streptococcus infection is prevalent. In the cages and ponds where Streptococcus related mortality was ≥ 10%, the Nile tilapia aquaculture was found to be profitable even if the amount paid for genetically selected Streptococcus resistant tilapia fingerlings was 100% higher than the amount paid for standard fingerlings.

## Introduction

Nile tilapia has become the most important freshwater aquaculture species in the last three decades with a global market value of US $7.9 billion in 2020^1^. In these three decades, the Nile tilapia industry has gone through tremendous changes in aquaculture practices; from extensive farming to the rapid expansion in intensive commercial farming^2^. With the increase in the intensification of production, disease outbreaks have been seen as the major threat to sustainable tilapia production worldwide.

Of all the mortality due to infectious diseases, one of the most important causative agents is the endemic bacterial disease Streptococcosis^3^, with losses being reported up to 70% (Trapia, personal communication). Streptococcosis become more likely when the water temperature rises above 30 °C^4,5^. In the current scenario of widespread global warming, the probability of heatwaves is increasing and with it the probability of Streptococossis as well. Until now the vast majority of the tilapia industry relies on the post-infection strategy of treatment with antibiotics, with few large-scale commercial farmers shifting to a pre-infection vaccination strategy to control the infection and reduce the mortality due to Streptococcosis^4^.

These methods have their limitations. It is common practice to treat the entire population of fish orally using antibiotics, even though only a small percentage of the fish are infected with Streptococcosis. This makes antibiotics therapy expensive and increases the concerns for anti-microbial resistance, both in fish and humans. Similarly, vaccination by intraperitoneal injection is possible only after the fish reaches a certain size, thereby vaccines have limited effectiveness in the early stages, which can have a huge economic implication if there is any disease outbreak during this phase. Furthermore, vaccinating large groups of fish is difficult and labour intensive. Hence alternative sustainable strategies to control the major diseases must be implemented in Nile tilapia aquaculture for animal health, welfare and productivity; without compromising food safety, environment, and human health.

The development of tilapia strains resistant to Streptococcosis has been proposed as the alternative long-term sustainable strategy to control the disease^6,7^. However, the economic analysis to weigh the different strategies for controlling Streptococcosis infection is lacking, with only one such study available for vaccination^8^. These financial evaluations allow farmers to choose the models of infection control based on economic profitability. Hence, the first aim of the paper is to assess the economic worth of using tilapia fingerlings tolerant to Streptococcosis in two major production environments: cage and pond production system of Nile tilapia. The second aim of the paper is to assess the profitability of paying the higher price for such tilapia resistant strains at different levels of infection.

## Methodology

We built a stochastic model to undertake a partial budget analysis of using genetically selected Nile tilapia fingerlings to control Streptococosis associated with *Streptococcus agalactiae* in the Nile tilapia industry. The model does not take time into account and considers the benefits and costs that are likely to occur in the new steady-state (one production cycle), as a result of the proposed intervention.

The partial budget model was developed using an Excel spreadsheet (Microsoft Corp., Redmond, WA, USA) and divided into four components, as proposed by Rushton^9^: (a) new revenues, (b) costs saved, (c) revenue foregone, and (d) new costs, that will be detailed in different subsections below. To assess the financial worth of such investment by farmers, we calculated the difference between benefits (a + b) and costs (c + d). If the marginal benefits exceed the marginal costs, then it would be advantageous for the farmer to invest in tilapia fingerlings resistant to Streptococcosis. The costs and benefits of using tilapia fingerlings resistant to Streptococcosis were calculated at the farm level for one tilapia production cycle. We considered two epidemiological scenarios based on the production system (cage and ponds).

In general, the Nile tilapia production system for large fish (i.e., fish weighing more than 800 g) is based on intensive farming practices and can be divided into two major categories; cage and pond farming system, based on the rearing place. In the cage farming system, tilapia are reared in cages suspended in lakes and reservoirs after the nursery period. Whereas, in a pond farming system, large ponds with aeration are used to rear the tilapia until harvest^10,11^. There is a major difference between these two farming systems in the adoption of vaccine technologies. While many cage farmers in Malaysia and Latin America have adopted vaccines to control Streptococcosis, there is limited adoption of vaccination by pond farmers^4,12^.

For each farming system, we used Pert distributions to account for the likelihood for the variability of the *Streptococcus* related mortality, relative percent survival, average feed conversion ratio, average weight of *Streptococcus* related mortality, average fish market price, the weight of treated fish, as well as genetics cost per fish.

The stochastic components of the model were handled with @Risk 7.5 (Palisade Corporation, NY, USA), an Excel add-in, using Latin Hypercube sampling, 10,000 iterations, and a random seed. The net return due to the use of the genetically selected Streptococcus resistant tilapia fingerlings was the outcome of the model. We did a sensitivity analysis of the input variables using the software’s built-in tool. Finally, we carried out a break-even (benefits ≥ costs) analysis for a combination of the cost of genetics and *Streptococcus* related mortality, given the two farming systems.

The values assigned to all modelled variables were based on information provided by the genetics company, hatcheries, producers, vaccine resellers, published data and personal communication from experts (Table 1) based on Malaysian tilapia farming conditions. The severity of Streptococcosis is observed to be more in cages than in the pond farming system, probably due to significantly higher density in cages^3^; which is modelled in the scenarios (Table 1). RPS data for pond and cage culture used to get the three Pert parameters (minimum, most likely, and maximum) is described in Table 2. One of the main reasons for the difference in the RPS values for the two-farming system is due to the difference in the practice of vaccination as stated previously.

**Table 1:** Parameters included with fixed values and with Pert probability distribution in the economic model of using tilapia fingerlings resistant to Streptococcosis.

With fixed values			
Parameter	Fixed value	Reference	
Batch size in number of fish	30,000	Hatcheries	
Average feed conversion ratio (FCR)	1.6	Producers	
Average stocking weight (kg)	0.03	Hatcheries	
Average harvest weight (kg)	1	Producers, Genetics company	
Feed price per kg	US$0.7	Producers	
Fingerling price	US$0.04	Hatcheries	
Cost of florfenicol per kg	US$242	Resellers	
Cost of vaccine dose per fish	US$0.02	Vaccine resellers	
Price of vaccine labour per fish	US$0.01	Vaccine resellers	

With Pert probability distribution			
Parameter	Minimum	Most likely	Maximum
RPS (cage)	8	20	31
RPS (pond)	16	32	52
FCR	1.45	1.52	1.6
*Streptococcus* related mortality (cage)	1%	8%	30%
*Streptococcus* related mortality (pond)	2%	4%	8%
Average weight of dead fish (g)	100	300	1000
Average weight of treated fish (g)	100	300	700
Average fish market price (US$)	0.96	1.2	1.44

**Table 2:** Different sources of information on relative percent survival (RPS) related to the use of genetically selected Nile tilapia fingerlings resistant to Streptococcosis in the pond and cage culture system.

Production system	Source	Models	Mortality (%)		RPS (%)	References
			Genetically selected fingerlings	Normal fingerlings		
Pond	Field trial	Trial 1	29.55	43.45	32	Unpublished results
		Trial 2	33.05	43.45	24	
	Experimental validation	IP infection model	28.67	49.67	42	^6^
		Cohab infection model	32.33	43	25	
	Literature reviews	Generation G0	66.5	99.5	33	^7^
		Generation G1	66.5	78.9	16	
		Generation G1	27.9	58.06	52	^19^
Cage	Field trial^a^	Trial 1	17.21	25.01	31	Unpublished results
		Trial 2	21.42	23.29	8	

Most probable value for the Pert distribution is calculated from the mean of all available RPS values in the Table. IP is intraperitoneal and cohab is cohabitation infection model.

^a^Both genetically selected and normal fingerlings were vaccinated during the experiment to mimic the cage culture practice of vaccinating the fish as stated in the text.

### Description of the partial budget model

#### New costs

Genetics-related costs comprise the unit price of *Streptococcus* resistant tilapia fingerlings discounted the price normally paid for standard fingerlings. The average price of the standard fingerling in Malaysia is $0.04 as reported by majors’ fingerling resellers and hatcheries. The unit cost of *Streptococcus* resistant tilapia fingerlings considers a variation of 10% to 30% over the amount paid for standard fingerling. This uncertainty was also modelled by a Pert distribution in the stochastic model (Table 1). The extra cost of *Streptococcus* resistant tilapia fingerlings was derived as:

$$ New \; costs  = [\left( {C_{Resistant} -C_{Standard} } \right) \times N_{Fish} ] $$

where N_Fish_ = number of fish in batch, C_Resistant_ = cost of *Streptococcus* resistant tilapia fingerlings (per fish), C_Standard_ = cost of standard tilapia fingerlings (per fish),

#### Revenue foregone

There is no revenue foregone because of *Streptococcus* resistant tilapia fingerlings.

#### Costs saved

Expenditure with antibiotics and feed intake are considered the only cost saved if *Streptococcus* resistant tilapia fingerlings are used. Genetically resistant fingerlings have the potential to contribute to reduced antibiotic usage due to the reduced prevalence of diseases^13^. Lower feed conversion ratio (FCR) and better growth in resistant fish populations compared to counterparts are expected. We then assumed a 50% reduction in antibacterial usage if *Streptococcus* resistant tilapia fingerlings are used when compared to a standard batch. The costs saved were calculated as follows:

$$ \begin{aligned} {\text{Costs saved }} & = \left[ {\left\{ {\left( {\left( {N_{Treat\_Resistant} \times N_{Treatments} } \right) \times Atb_{Dose} \times \, 50\% } \right) \, - \, \left( {N_{Treat\_Standard} \times N_{Treatments} \times Atb_{Dose} } \right)} \right\} \times Atb_{Price} } \right] \hfill \\ & \quad + \left[ {\left( {BioH_{Standard} \times \, FCR_{Standard} } \right) \, - \left( {BioH_{Resistant} \times \, FCR_{Resistant} } \right)} \right] \hfill \\ \end{aligned} $$

where N_Treat_Resistant_ = expected biomass of *Streptococcus* resistant tilapia to be treated with antibiotics, N_Treat_Standard_ = expected biomass of standard to be treated with antibiotics, N_Treatments_ = number of antibacterial treatments over the production cycle, Atb_Dose_ = dose of the antibacterial used, Atb_Price_ = cost of antibacterial per kg, *BioH*_*Standard*_ = expected biomass of standard harvested (in kg), FCR_Standard_ = feed conversion ratio for standard, *BioH*_*Resistant*_ = expected biomass of resistant harvested (in kg), and Resistant = feed conversion ratio for resistant fish. Antibacterial treatment was considered using florfenicol (20 mg/kg body weight/day) for ten consecutive days (Merck Animal Health, USA). Considering the difficulty in calculating the exact time of treatment, we calculated N_Treat_Resistant_ assuming survival fish for each scenario (Resistant *vs* Standard) and multiplying it by the average weight of treated fish, which was also modelled by a Pert probability distribution (Table 1).

#### New revenue

An increase in fish survival is the only consequence of using genetically resistant fingerlings that yields an additional return. It was calculated as:

$${\text{New revenue }} = \left[ {\left( {Survival_{{Resistant}}  - {\text{ }}Survival_{{Standard}} } \right){\text{ }} \times {\text{ }}W_{{Harvested}}  \times {\text{ }}Fish_{{Price}} } \right]$$

Considering:

$$ {\text{Survival}}_{{{\text{Standard}}}} = N_{Fish  } - MortStrep_{Standard} $$

$$ {\text{Survival}}_{{{\text{Resistant}}}} = N_{Fish} -  MortStrep_{Resistant} $$

$$ {\text{Mortality}}_{{{\text{Resistant}}}} = Mortality_{Standard} \times \left[ {1 \, - \, RPS} \right] $$

where Mortality_Standard_ = total mortality due to Streptococcosis in a farm rearing standard fingerlings (number of fish), Mortality_Resistant_ = total mortality due to disease in a farm rearing genetically *Streptococcus* resistant fingerlings (number of fish), MortStrep_Standard_ = expected mortality due to *S. agalactiae* if standard fingerlings (in %), MortStrep_Resistant_ = expected mortality due to *S. agalactiae* if genetically *Streptococcus* resistant fingerlings (in %), RPS = relative percent survival provided by genetically *Streptococcus* resistant fingerlings (in %), W_Harvested_ = fish weight during harvesting and Fish_Price_ = fish market price. *Streptococcus related* mortality and RPS were modelled using Pert probability distribution (Table 2).

## Results

**Figure 1:**
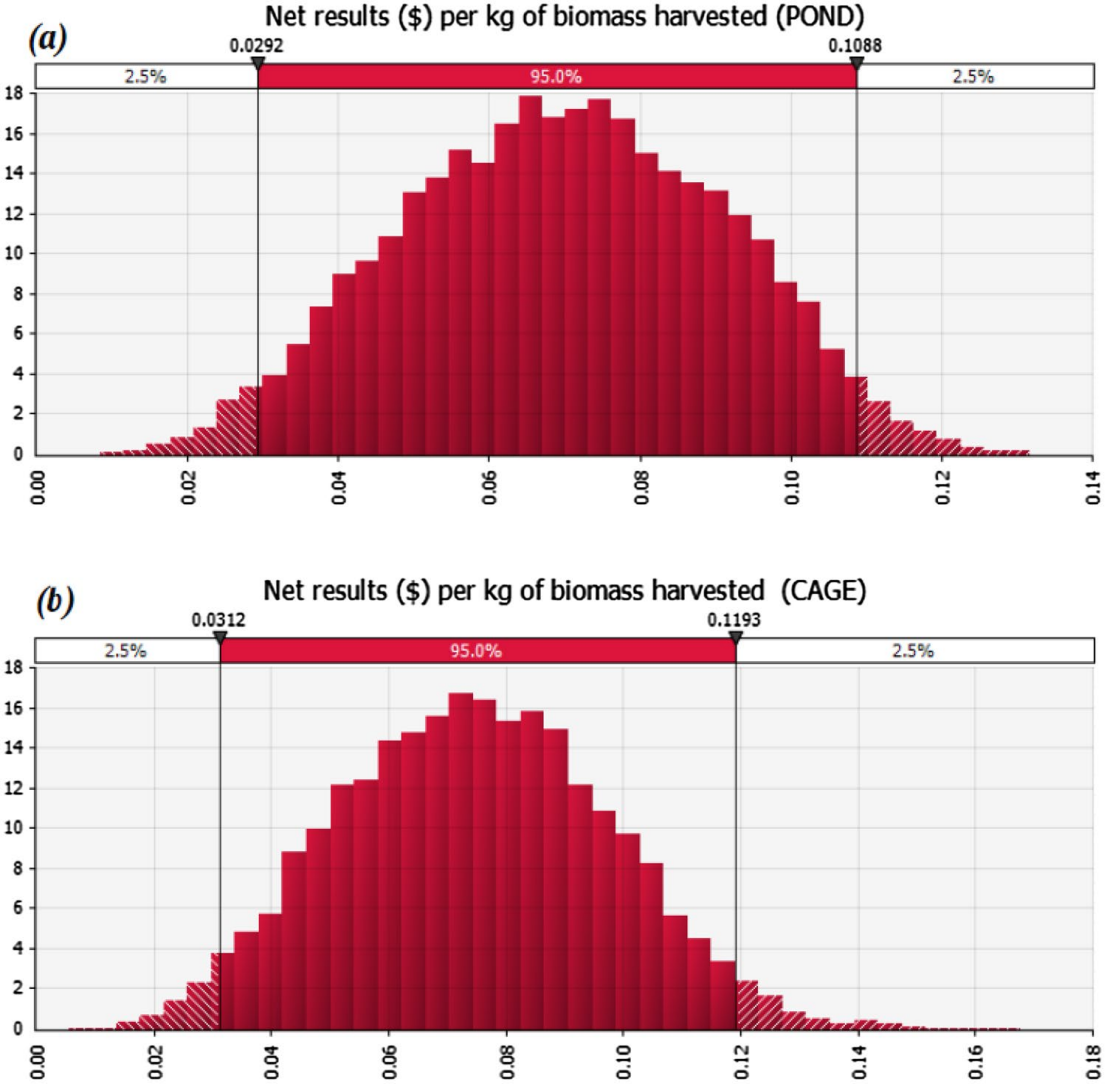
Probability distribution of net result per kg of biomass harvested for (**a**) pond scenario and (**b**) cage scenario. The histogram shows the range of possible outcomes (x-axis) and their relative likelihood of occurrence (y-axis).

The results for the two different production system scenarios where we used probability distributions to model variability and uncertainty are shown in Fig. 1. Figure 1 shows the range of possible outcomes (x-axis) and their relative likelihood of occurrence (y-axis). Because a simulation yields many possible values for the outcome, the summary statistics is used to summarize the range of net results per kilogram of biomass harvested when using tilapia strains resistant to *Streptococcosis* at the farm level. For tilapia pond farms, the average net result is US$ 0.07 (± 0.021) but it can range from US$ 0.01 to US$ 0.13 depending on several inputs (and their uncertainty) that interact to produce the outcome. Similarly, for tilapia cage farms, the average net result is US$ 0.075 (± 0.023) but it can range from US$ 0.01 to US$ 0.17.

Table 3 displays the layout of the baseline partial budget model used to estimate profitability of using genetically *Streptococcus* resistant fingerlings in a modal pond tilapia farm in Malaysia, for one production cycle starting with 30,000 fingerlings, 15% mortality over the whole period, improvement in FCR (1.60 for standard and 1.52 for resistant), which is a very realistic scenario, genetics-related costs (price normally paid for standard fingerlings + 20%) and an RPS of 32%. The net change in income (benefits – costs) was US$ 0.12 per kilogram of biomass harvested. A similar deterministic approach (Table 3) was used for a modal cage tilapia farm in Malaysia, for one production cycle starting with 60,000 fingerlings, 20% mortality over the whole period, a similar improvement in FCR (1.60 for standard and 1.52 for resistant), genetics-related costs (price normally paid for standard fingerlings + 20%) and an RPS of 20%. The net change in income (benefits – costs) was US$ 0.11 per kilogram of biomass harvested.

**Table 3:** Partial budget for genetically selected Nile tilapia fingerlings resistant to Streptococcosis, considering one production cycle of tilapia farming in the pond system (starting with 30,000 fingerlings) and the cage system (starting with 60,000 fingerlings) using deterministic approach.

			Pond			Cage		
			No	Cost/unit	Total	No	Cost/unit	Total
Costs (US$)	New costs	Genetically selected fingerlings	30,000	0.01	300	60,000	0.01	600
	Revenue foregone	–	–	–	–	–	–	–
Total costs (US$)					300			600
Benefits (US$)	Costs saved	Reduction in antibiotic usage (kg)	1.4	242.4	350	2.74	242.4	663
		Feed (kg)	2040	0.7	1428	3840	0.7	2688
	New revenue	Extra fish sales	1440	1.2	1728	1800	1.2	2880
Total benefits (US$)					3506			6231
Net result per production cycle due to genetics (total benefits-total costs) (US$)					3206			5631
Net result per kilogram of biomass harvested due to genetics (US$)					0.12			0.11

The results for the two different scenarios where we used probability distributions to model variability and uncertainty are shown in Table 4 and displayed as a break-even analysis (i.e. the probability of benefits being equal or greater than costs for each combination of variables). The extra cost of genetically selected *Streptococcus* resistant fingerlings and cumulative *Streptococcus* related mortality are the only parameters that can be realistically assessed by the farmer before deciding whether to buy or not genetically selected *Streptococcus* resistant tilapia fingerlings and therefore were the parameters chosen for the break-even analysis. In the pond scenario, when *Streptococcus* related mortality is over 20% the net return would break even in 100% of iterations, indicating that using *Streptococcus* resistant fingerlings is profitable, even when the extra cost paid is 100%. In scenarios with lower expected mortality, profitability is more dependent on the extra amount paid. For example, if the expected *Streptococcus* related mortality is 1% and the extra cost paid for genetics is 50%, the probability of break-even drops to 46.2%. For the cage scenario, the break-even probability was more dependent on expected *Streptococcus related* mortality. Either way, if the extra amount paid is up to 30%, the profitability is very likely to occur even in low *Streptococcus* related mortality (≥ 5%).

**Table 4:** Probability of breaking-even (benefits ≥ costs) for a combination of cost of genetics and *Streptococcus* related mortality, given two production system scenarios.

Cost	POND					CAGE				
	Mort					Mort				
	1%	5%	10%	15%	≥ 20%	1%	5%	10%	15%	≥ 20%
10%	**100%**	**100%**	**100%**	**100%**	**100%**	**100.0%**	**100%**	**100%**	**100%**	**100%**
20%	**100%**	**100%**	**100%**	**100%**	**100%**	**99.7%**	**100%**	**100%**	**100%**	**100%**
30%	**94.6%**	**100%**	**100%**	**100%**	**100%**	87.6%	**100%**	**100%**	**100%**	**100%**
40%	73.2%	**100%**	**100%**	**100%**	**100%**	63.2%	**98.2%**	**100%**	**100%**	**100%**
50%	46.2%	**100%**	**100%**	**100%**	**100%**	36.7%	**90.3%**	**100%**	**100%**	**100%**
60%	23.0%	**95.1%**	**100%**	**100%**	**100%**	16.5%	67.8%	**97.5%**	**100%**	**100%**
70%	8.2%	83.6%	**100%**	**100%**	**100%**	4.2%	41.5%	**91.7%**	**98.9%**	**100%**
80%	1.2%	64.5%	**99.7%**	**100%**	**100%**	0.3%	20.1%	80.1%	**96.6%**	**100%**
90%	0%	42.1%	**96.9%**	**100%**	**100%**	0%	7.0%	61.0%	**92.0%**	**98.4%**
100%	0%	22.5%	**91.2%**	**99.8%**	**100%**	0%	1.6%	37.5%	81.7%	**96.0%**

The bold is used to highlight the 90% probability of break-even. “Mort” indicates mortality due to Streptococcosis and “Cost” indicates the extra cost of genetically selected Nile tilapia fingerlings resistant to Streptococcosis over the standard fingerlings.

**Figure 2:**
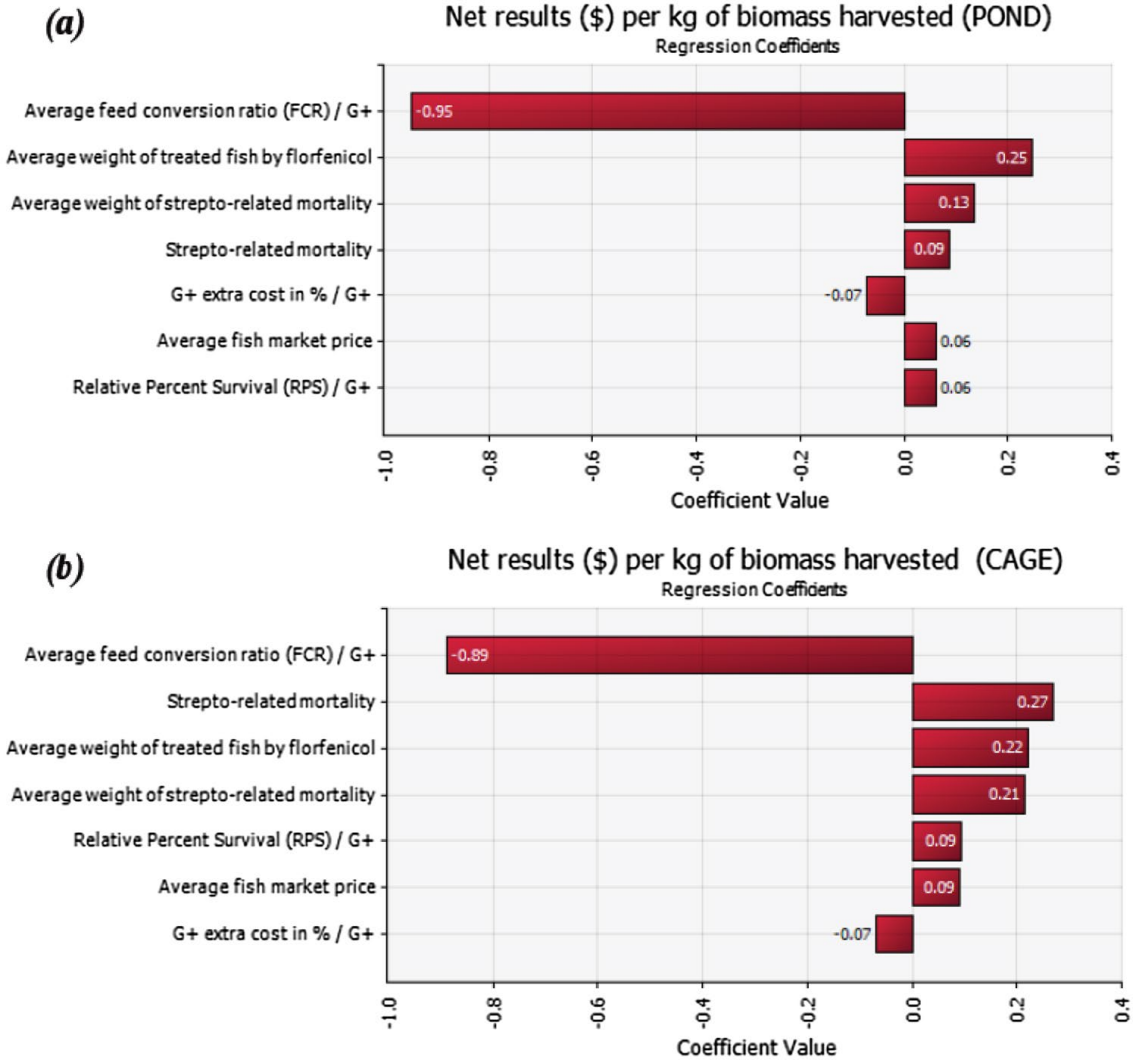
Regression sensitivity (tornado plot) for variables modelled with Pert distributions for the (**a**) pond scenario and (**b**) cage scenario. G+ indicates genetically selected Nile tilapia fingerlings resistant to Streptococcosis.

Figure 2 shows the results from the regression sensitivity analysis for pond and cage farms, respectively, displaying a ranking of the parameters that impact the output: net results per kg of biomass harvested. Parameters with the largest impact on the distribution of the output have the longest bars in the graph and are ordered from top-down. A positive coefficient indicates that this input has a positive impact: increasing this input will increase the output. A negative coefficient indicates that this input has a negative impact: increasing this input will decrease the output. For both production systems, the results suggest that “Average FCR” had the greatest impact (negative) in the net results per kg of biomass harvested, being slightly higher for pond (− 0.95) when compared to cages (− 0.89). For pond farms, the second most influential input variable was “Average weight of treated fish by florfenicol” followed by “Average weight of *Streptococcus* related mortality”. On the other hand, for cage farms, the second most influential input variable was “*Streptococcus* related mortality” followed by “Average weight of treated fish by florfenicol”, and “Average weight of *Streptococcus* related mortality”. The inputs “Genetically selected fingerlings extra cost”, “RPS”, and “Average fish market price” had minor effects on the output, in both scenarios.

## Discussion

Streptococcosis has become the major bacterial disease affecting commercial tilapia farming worldwide and various prevention and control measures are being applied affecting the profitability of the farms^4^. Using genetics to control disease in aquaculture has become a common practice^14^, which is because breeders were able to reduce the incidence of a major viral disease, infectious pancreatic necrosis (IPN), to near zero in salmon farming via selective breeding^15^. The power of genetics to control Streptococcosis is increasingly being recognised^6,7^ with various breeding programs incorporating this trait in their selective breeding program^16^ and *Streptococcus* resistant tilapia fingerlings being made available to the farmers^17^.

Our results indicate that using genetically selected fingerlings is likely to be profitable in Nile tilapia farms where Streptococosis is a production constrain. For either pond or cage tilapia farms, genetics proved to be profitable in scenarios where *Streptococcus related* mortality are higher (≥ 15%), even though the cost to be paid was close to 100% higher than the cost of a standard fingerling.

In the pond scenario, the net return was positive (benefits ≥ costs) in 99.7% of iterations, indicating that genetically selected *Streptococcus* resistant fingerlings are profitable, even in the absence of any improvement in feed conversion, which is a very conservative assumption. However, the profitability of genetically selected *Streptococcus* resistant fingerlings can be lower when cumulative *Streptococcus* related mortality is lower (< 10%), in which case it would be more dependent on the extra cost to be paid for the genetically selected *Streptococcus* resistant fingerlings. For example, genetically selected *Streptococcus* resistant fingerlings is likely to yield economic gains for pond production when the extra cost of genetically selected *Streptococcus* resistant fingerlings is up to 30% of the price paid for the standard fingerling, regardless of *Streptococcus related* mortality (in at least 94.6% of iterations). Whereas in ≥ 10% *Streptococcus related* mortality genetically selected *Streptococcus* resistant fingerlings is very likely (at least 91.2% of iterations) to be lucrative even if the amount paid for genetically selected *Streptococcus* resistant fingerlings was 100% higher than the amount paid for standard fingerlings.

In the cage scenario, despite likely being profitable, *Streptococcus* related mortality had a greater impact on the net results compared to pond farms, as shown by the sensitivity analysis. Thus, the profitability of genetically selected *Streptococcus* resistant fingerlings is lower when cumulative mortality was lower, in which case it would be more dependent on the genetics related costs. Likewise, Thorarinsson and Powell^18^ and Delphino et al.^8^ described disease risk level (mortality pre-intervention) to have a profound influence on the profitability of salmon and tilapia vaccination, respectively. In addition, in cage farms where *Streptococcus* related mortality is very low, the profitability of the use of genetically selected *Streptococcus* resistant fingerlings would be very dependent on better “FCR” and “genetics related cost”. The inputs “fish market price” and “RPS” had minor effects on the output, in production system scenarios.

This model considered marginal benefits and costs that are directly associated with the use of genetics to control Streptococcosis in tilapia farms and was not designed to be a farm budget. Although our results indicate that genetically selected *Streptococcus* resistant fingerling is likely to yield economic gains, tilapia farmers should be aware that the profitability of the intervention is a combination of parameters. Therefore, the baseline partial budget model must be updated to reflect changes in economic (e.g., market price, genetics price) or biological factors (e.g., RPS and FCR). Although this model is based on conservative values and considers uncertainty about the modelled parameters, we conclude that the use of genetically selected *Streptococcus* resistant fingerlings is likely to be profitable in Nile tilapia farms, under similar economic and biological factors assessed.

## Conclusions

In both ponds and cage production systems of Nile tilapia, the use of genetically selected *Streptococcus* resistant tilapia fingerlings was found to be profitable where *Streptococcus* infection is prevalent. Higher the mortality due to *Streptococcus* infection, higher the economic profitability of using such *Streptococcus* resistant tilapia fingerlings. In the cages and ponds where *Streptococcus* related mortality was ≥ 10%, the Nile tilapia aquaculture was found to be profitable even if the amount paid for genetically selected *Streptococcus* resistant tilapia fingerlings was 100% higher than the amount paid for standard fingerlings.

## Data Availability

All the parameters and data used for the analysis are listed in the manuscript.
